# Effects of a treadmill and oculomotor dual-task intervention vs. -nordic walking on balance in Parkinson’s disease patients − a pilot study

**DOI:** 10.1016/j.prdoa.2025.100392

**Published:** 2025-09-08

**Authors:** Marc Niering, Corinna Wirth, Rainer Beurskens, Elisa Ueding, Tim Fischer, Johanna Seifert

**Affiliations:** a Institute of Biomechanics and Neurosciences, Nordic Science, Hannover, Germany; b Triagon Academy Munich, School of Sports, Psychology and Education, Ismaning, Germany; c Department of Health and Social Affairs, FHM Bielefeld - University of Applied Sciences, Bielefeld, Germany; d Institute of Sports Science, Faculty of Humanities, Leibniz University Hannover, Hannover, Germany; e Exercise Science & Neuroscience Unit, Department of Exercise & Health, Paderborn University, Paderborn, Germany; f Department of Psychiatry, Social Psychiatry and Psychotherapy, Hannover Medical School, Hannover, Germany

**Keywords:** Parkinson’s disease, Balance, Oculomotor function

## Abstract

**Background:**

Oculomotor function has been established as a critical factor influencing balance, and both domains are frequently impaired in Parkinson’s disease (PD). Despite increasing recognition of younger PD cases, targeted therapeutic research remains limited.

**Purpose:**

This pilot study evaluated the effects of a treadmill-based oculomotor dual-task intervention in individuals with PD, comparing its impact on balance and non-motor outcomes with that of Nordic walking (NW).

**Method:**

Using a sequential within-subjects design, this study contrasts a four-week NW intervention with a subsequent four-week oculomotor intervention. Baseline, interim, and post-intervention assessments were conducted, with data collected using both qualitative and quantitative measures.

**Results:**

The treadmill and oculomotor intervention led to greater improvements in unilateral and static balance parameters compared to NW, particularly under horizontal eye-movement conditions. While motor scores improved comparably in both interventions, non-motor domains such as emotional well-being and stigma improved only after the oculomotor protocol.

**Conclusion:**

Treadmill-based oculomotor dual-task training appears to be a viable and time-efficient alternative to NW for improving specific aspects of balance and non-motor function in PD. Given its indoor feasibility and short duration, this approach may enhance accessibility in clinical and home-based rehabilitation settings.

## Highlights

• Treadmill-oculomotor dual-task intervention improved unilateral balance in early-onset PD.
• Compared to Nordic walking, oculomotor training enhanced non-motor and affective domains.
• Static balance improved significantly under oculomotor dual-task conditions.
• Visuo-motor coordination may represent a critical target in early-onset Parkinson’s therapy.

## 1 Introduction

Parkinson's disease (PD) is one of the most common neurodegenerative disorders today[1,2]. The condition is characterized by selective degeneration of dopaminergic neurons in the substantia nigra pars compacta[3], leading to dopamine deficiencies that impair movement control[4]. Since 1990, the prevalence of PD has more than doubled[5], and further increases are projected for the coming decades[4,6]. PD diagnosed before the age of 70 accounts for a substantial portion of cases and includes patients who, while not classified as early-onset, still face prolonged disease durations with progressively debilitating symptoms[7,8]. In addition to the primary motor symptoms tremor, rigidity, and akinesia[4], balance impairments are particularly impairing in PD. Although pharmacological interventions like levodopa may temporarily alleviate symptoms, they often fall short in addressing balance deficits[9,10]. Therefore, there is an urgent need for effective, non-pharmacological treatments targeting both motor and non-motor symptoms in early-onset PD.

Nordic walking (NW) has been extensively employed as a balance intervention, enhancing stability through rhythmic, coordinated movements of both the upper and lower body[11,12]. However, the underlying causes of balance impairments in PD extend beyond motor deficits and may involve oculomotor dysfunction. Studies reveal that eye movement and visual system deficits are highly correlated with balance impairments in PD, suggesting an integral role of oculomotor control in balance[13,14]. Given the neurophysiological connections between the oculomotor and vestibular systems, oculomotor exercises may therefore provide an innovative approach to improving balance through enhanced visual-motor coordination[15,16]. The effectiveness of visual skills training in neurorehabilitation has been demonstrated in other conditions as well, where targeted visual exercises led to measurable improvements in cognitive and executive functions[17]. In dual-task studies, Beurskens et al. [18] demonstrated that cognitive demand impedes postural control, indicating that dual-task balance interventions may help PD patients manage the increased cognitive load in everyday tasks, thus enhancing postural stability and reducing fall risk. This evidence supports the potential of oculomotor dual-task interventions to induce neuroplastic adaptations beneficial for PD management.

Despite the relevance of this relationship, most studies on PD focus on older patients (average age > 70 years), who may face additional complications such as cognitive decline or dementia[9,19].

In this study, we implemented a sequential within-subjects design to compare a treadmill-based oculomotor dual-task intervention (ALT) with a conventional NW intervention (CON). We hypothesized that ALT would provide a more efficient, time-saving, and weather-independent alternative to NW, with potential benefits extending to non-motor outcomes through improved functional connectivity between the visual and limbic systems. By exploring the effects of ALT on both motor and non-motor domains, this study aims to address a critical gap in treatment strategies for PD. Our findings hold potential implications for both clinical practitioners and researchers aiming to develop comprehensive treatment models for neurological disorders involving balance dysfunctions.

## 2 Methods

### 2.1 Study design

**Fig. 1:**
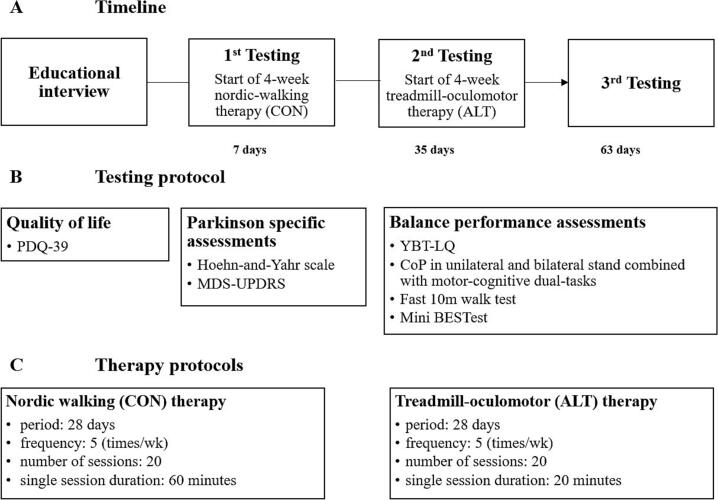
A-C. Illustration of the study design, testing procedures, and interventions. *Note.* CON: Conventional Nordic walking therapy; ALT: Alternative treadmill-oculomotor therapy; PDQ-39: Parkinson’s Disease Questionnaire; MDS-UPDRS: Movement Disorder Society-sponsored revision of the Unified Parkinson’s Disease Rating Scale; YBT-LQ: Y-Balance Test − Lower Quarter; CoP: Center of Pressure; Mini BESTest: Mini Balance Evaluation Systems Test; wk: week.

To compare CON with ALT, a sequential within-subjects design was applied in a case series, as schematically illustrated in Fig. 1A-C. This design was chosen due to the novelty and complexity of the intervention protocol, which to our knowledge has not yet been implemented in this form. Moreover, the study was intended as a feasibility pilot for a larger trial, and the inclusion of PD patients between 41 and 69 years of age, thus below the average age range typically seen in PD trials, allowed for a clearer attribution of intervention effects without confounding age-related or cognitive comorbidities.

Importantly, the initial assessment and intervention phases were not conducted simultaneously for all participants. Participant #1 completed all testing sessions and both interventions individually, approximately ten months prior to the remaining participants, in order to assess the feasibility of the protocol and refine procedural details. The remaining eight participants were subsequently tested and trained during the following summer period.

Seven days before the initial assessment (T1), the study procedures were thoroughly explained to the participants in an educational session, which included practice sessions of all test exercises to reduce performance anxiety and ensure accurate execution. While three of the participants were already familiar with NW, they had not engaged in it regularly, and the oculomotor dual-task exercises (specific to ALT) were entirely novel and complex. This prior familiarization was thus essential to prevent cognitive overload from unfamiliar tasks during testing and to optimize training efficacy through motor learning [20]. In addition, the Morningness-Eveningness Questionnaire (MEQ)[21]was administered to determine the optimal testing time based on the participants’ chronotype and self-reported symptom patterns. Eight participants were classified as 'morning types', and one as an 'intermediate type' based on MEQ scores. All assessments were conducted in the morning hours, which aligned with participants' self-reported symptom profiles and is consistent with typical hormonal and dopaminergic fluctuations in PD. Symptom severity in PD often increases in the evening due to reduced cortisol and adrenaline levels[22].

All testing sessions were conducted in summer, ensuring consistent daylight, as light exposure is known to influence dopaminergic function, which may, in turn, impact both visual and motor performance[23]. Furthermore, studies link exposure to daylight with improved mood, potentially affecting motor behavior[24]. The protocol included three testing sessions (T1-T3) with identical procedures for qualitative and quantitative data collection. The first session (T1) was followed by a four-week NW intervention, after which the second testing session (T2) was completed. This was immediately followed by the four-week ALT intervention, culminating in the final session (T3), as illustrated in Fig. 1A. The intervention period of four weeks per phase was chosen based on prior evidence indicating that clinically meaningful improvements in motor and dual-task outcomes can be achieved within this timeframe[11,25].

### 2.2 Participant characteristics

Nine participants with PD were enrolled in the study (2 females, 7 males; mean age 59.2 ± 8.1 years; disease duration 7.3 ± 1.0 years). Baseline characteristics are summarised in Table 1.

**Table 1:** Participant characteristics at baseline.

Characteristic	#1	#2	#3	#4	#5	#6	#7	#8	#9	Overall Mean ± SD
Sex [F/M]	M	M	F	F	F	M	M	M	M	2F (22.2 %) / 7 M (77.8 %)
Age [years]	41	57	63	59	55	60	55	64	69	59.2 ± 8.1
Disease duration [years]	4	8	7	8	7	7	8	8	9	7.3 ± 1.0
Body height [cm]	170	175	155	171	178	173	182	169	175	171.6 ± 6.8
BMI	23.8	24.1	28.9	24.2	25.3	27.3	22.2	31.3	26	25.3 ± 3.0
Body fat	13.7	20.3	30.5	25.6	24.8	20.1	25	32.1	21.7	24.4 ± 5.7
Levodopa [mg/day]	600	400	350	400	500	600	450	400	450	466.7 ± 101.4
MDS-UPDRS Total	40	69	57	67	54	76	55	60	84	63.6 ± 12.7
Modified Hoehn-and-Yahr-Scale	1.5	2.0	1.5	2.0	2.5	2.5	2.0	1.5	2.0	2.0 ± 0.3

No formal a priori power analysis was conducted, as this investigation was designed as a pilot study within a longer‑term recruitment process. The present sample therefore reflects the participants who had completed all testing and both intervention phases at the time of analysis. The study by Bang and Shin [11], which reported a large effect size (*d* = 0.74) for improvements in the fast 10‑Meter‑Walk Test (10MWT) when comparing NW to treadmill training in patients with PD, was considered when reflecting on the sample size requirements for future trials. Based on this effect size and assuming a repeated‑measures design with *α* = 0.05, power = 0.80, and a conservative correlation of *r* = 0.5, approximately n = 17 participants would be required. The current sample of n = 9 therefore provides preliminary estimates of intervention effects but is underpowered for confirmatory statistical testing.

One participant (#1) met the criteria for early‑onset PD, having been diagnosed at the age of 37. Disease severity ranged from Hoehn and Yahr stage 1.5 to 2.5 (mean 2.0 ± 0.3), corresponding to mild to moderate motor impairment.

The mean daily levodopa equivalent dose was 466.7 ± 101.4  mg/day. Primary motor symptoms were asymmetrical in most participants, and all reported stable medication regimens throughout the study period. With the exception of participant #9, all participants were engaged in part‑time or full‑time employment at the time of data collection.

Absence of visual impairments was an inclusion criterion, as was the ability to walk independently on both a treadmill and level ground. All PD diagnoses were clinically confirmed by neurologists, with verification through medical documentation. Two participants (#5 and #7) reported a family history of PD, although none had undergone genetic testing. Participants were recruited from various neurology practices and clinics.

Prior to study inclusion, an interview was conducted to confirm eligibility criteria and to assess independent walking ability on the treadmill and on level ground. This eligibility assessment was carried out by the first author (MN).

### 2.3 Test procedures

#### 2.3.1 General administration guidelines

Before detailing the individual test procedures, the general protocol implementation rules are described. As balance performance can be influenced by multiple factors such as sleep quality, diet, waking time, time of day, and caffeine intake[26,27,28], participants were instructed to follow standardized preparatory conditions before each test session. These recommendations began on the evening prior to testing and included following a regular sleep schedule, consuming a balanced evening meal, and avoiding food and caloric beverages for at least three hours before bedtime to minimize metabolic influences on performance.

In addition, participants were advised to avoid bright artificial light exposure (e.g., television, computer, or mobile phone screens) for at least three hours before sleep, to keep their sleeping environment quiet and dark, and to maintain a comfortable room temperature. On the test day, participants were asked to refrain from caffeine intake in the morning and to consume only water before testing. All testing sessions were conducted in the morning, ensuring that participants were in their regular medicated “on” phase.

Sports science students measured height and weight to calculate body mass index (BMI), with body fat assessed using skinfold calipers.

#### 2.3.2 Assessment of quality of life

To assess the patients’ quality of life, the PDQ-39, a standardized and highly reliable (ICC = 0.34–0.96)[29]and valid instrument for PD patients[30]was administered. This self-reported questionnaire consists of 39 items covering domains such as mobility, social support, cognition, communication, and emotional well-being. The PDQ-39 yields a total percentage score, where lower scores reflect a better quality of life and higher scores indicate a more compromised quality of life. This assessment captures functional changes associated with quality of life, highlighting specific areas where the patient experiences the greatest impairment[31]. To prevent potential influences from the family environment, the patient completed the questionnaire on-site, in a private setting.

#### 2.3.3 Parkinson specific assessments

Subsequently, the subjects’ motor skills were assessed using PD-specific evaluation procedures in all test sessions (T1-T3). First, a physician classified the subjects according to the Hoehn and Yahr scale to determine PD severity. This internationally utilized scale captures the primary motor impairments characteristic of PD, with higher scores correlating with increased motor dysfunction and decreased quality of life[32,33].

Following this, the MDS-UPDRS was applied. This scale is recognized for its high reliability (ICC = 0.79–0.93) and validity in measuring disease progression and evaluating both the presence and severity of PD-related issues[34,35]. The German version of the MDS-UPDRS was administered by a physician, adhering strictly to the standardized protocol. The scale comprises four parts: the first assesses daily living experiences and non-motor aspects, while the remaining three focus on motor aspects. These motor sub-scores reflect dopaminergic function and show significant correlations with specific oculomotor parameters[13,36]. Lower scores denote better motor function in this context[34].

Additionally, the Mini Balance Evaluation Systems Test (Mini-BESTest) was conducted according to standard guidelines. This test, which demonstrates high reliability (ICC = 0.92), provides a qualitative assessment of fall risk and balance[37]. It evaluates performance across 14 different balance tasks, such as walking with body rotation or navigating obstacles, scoring each task from zero to two, with higher scores indicating better balance function[38].

#### 2.3.4 Measures of proactive balance performance

To assess proactive balance performance, the Y-Balance Test-Lower Quarter (YBT-LQ) was administered[39]. The YBT-LQ is a standardized, coordinative, and reliable test of lower-extremity postural control (ICC = 0.85–0.91)[40]and has also been shown to measure the effects of training interventions[41,42]. Inadequate coordination between sensory and motor components of the neuromuscular system may manifest as postural instability or uncoordinated movements, which are observable during this test[39].

For this study, a specialized YBT-LQ test kit was used. The patients stood on a single leg, centered along a marker line on a standing plate, and extended the opposite leg as far as possible in the anterior, posteromedial, and posterolateral directions. Each direction was tested three times per leg, with one side completed before switching. Between trials, the free foot was returned next to the standing foot. If the patient’s heel lifted or balance was lost, the trial was disregarded and repeated[39]. The highest score achieved in each direction was recorded[43].

While hand positioning has been shown not to significantly impact results[41], the patients performed the test with hands placed on the hips for consistency. Test scores were then adjusted relative to leg length to calculate a composite score for each leg[43].

#### 2.3.5 Measures of dynamic steady-state balance performance

Dynamic steady-state balance performance was assessed using the 10MWT, a validated and highly reliable measure (ICC = 0.99)[44]. Widely employed for measuring gait speed (m/s), this test is effective in demonstrating the impact of therapeutic interventions[45]. The participants were instructed to walk at a quick pace, as recommended by Lindholm et al. [45], with the test conducted once according to standardized guidelines. After a verbal start signal, time was recorded using a Samsung Galaxy Watch 2 (Samsung, Suwon, South Korea).

#### 2.3.6 Measures of static steady-state balance performance

To assess static steady-state balance performance, two adjacent 40 x 60 cm Kistler force plates (Kistler, Winterthur, Switzerland) were used to measure medio-lateral and anterior-posterior sway, total center of pressure (CoP) displacement, and ground reaction force. Dual force plates were employed in accordance with recommendations for assessing neurodegenerative conditions, as using a single plate may limit the evaluation of extremity asymmetries[46]. Force platform measurements are known for their high reliability (ICC = 0.78–0.92) and validity in diagnosing balance disorders in PD patients[47].

The patients were instructed to adopt a natural standing position on the force plates with arms relaxed at the sides. To standardize foot placement and ensure measurement consistency, the patients’ stance were set at a heel-to-heel distance of 325 mm, within the recommended range of 200–500 mm[48]. This distance was marked with adhesive tape on the plates to maintain consistency across all bilateral tests. Static steady-state balance was quantified by analyzing the total displacement of the CoP[49]. All assessments were conducted barefoot, with unilateral tests beginning on the participants' subjectively non-dominant leg. Ten visuo-spatial load conditions were applied, first bilaterally and subsequently unilaterally, to reveal any asymmetrical deficits[50]. The single-leg stance, a task simulating everyday motor activities (e.g., stair climbing), contributed to a total of 20 tests[51].

Each test was performed three times, with a 15-second pause between trials, during which the participants stepped down from the plate and then resumed the testing position. The average of the three trials was recorded as the final result. The 15-second pause also applied between the 20 distinct test conditions, ensuring a continuous sequence of trials. No additional rest was given between left-to-right leg transitions, with the participants switching legs directly after each task. For unilateral tasks, the participants were instructed to keep the raised leg relaxed at ankle height. Table S1 outlines the specific test conditions. For each condition, the participants were required to maintain a stable stance for ten seconds before data collection began, with each oculomotor task repeated ten times per direction.

#### 2.3.7 Visuo-spatial load conditions

Both exercises and balance assessments were supplemented with dual-tasks to represent various visuo-spatial load conditions (Table S1). Such dual-tasks are commonly applied to evaluate motor function in PD patients[52]and assess static steady-state balance performance under additional cognitive load. Postural disturbances in PD result not only from dopaminergic and cholinergic dysfunctions but also from impaired integrative sensory processing[53]. To investigate effects on postural control, visual dual-tasks were selected due to their pronounced impact on the postural control system[54,55].

The visual system plays a critical role in maintaining balance, working closely with the balance control system to convey essential environmental information[56]. Visual input is integral for maintaining the body’s center of gravity[57,58] and provides complex sensory information to the central nervous system (CNS), where it is processed and integrated [59]. This integration results in postural adjustments and compensatory movements, which are measurable via force plate analysis[56].

Furthermore, Zhang et al. [14] observed correlations between oculomotor changes and both motor and non-motor PD symptoms, a finding particularly relevant given the frequency of oculomotor deficits in PD patients compared to age-matched healthy controls [60,61]. These deficits may impair visual processing, as demonstrated in prior studies[62]. Finally, as eye movements are independently executed and arise from distinct cerebral regions[16], both horizontal and vertical directions were tested to enable a comprehensive assessment of oculomotor-specific deficits. Only horizontal and vertical movements were employed to reduce task complexity and maintain methodological consistency, as diagonal movements would introduce additional coordination demands and potentially confound the primary visuo-spatial load assessment.

*Visual deprivation*. Balance in the standing position was assessed under both eyes-open and eyes-closed conditions. Closing the eyes reduces visual system influence on balance, providing a more precise assessment of postural stability and the vestibular system's role in maintaining balance[63,64]. Testing and training balance without visual input have shown positive effects on gait in PD patients, highlighting the adaptive potential of other sensory systems[65].

*Smooth-pursuit eye movements.* Smooth-pursuit eye movements were included due to their significant impact on balance [59]. These movements were tested by positioning the patients 0.6 m in front of an 85-inch LCD TV (screen dimensions: 189 cm width, 106 cm height; Samsung, Suwon, South Korea) displaying a three-centimeter black dot on a white background that moves horizontally and vertically using Microsoft PowerPoint. Prior to testing, the patient’s maximum viewing distance was recorded to calibrate the setup.

For each participant, the dot moved horizontally up to 90 % of their individually assessed maximum viewing distance, corresponding to a lateral displacement of approximately 90  cm from the screen center. Vertically, the dot traveled up to 70 % of each participant’s individual vertical visual range, equating to approximately 50  cm above and below the center. The target velocity for smooth-pursuit eye movements was set at 35°/s in both directions, based on the individualized dot trajectory and calculated motion speed, as recommended by Krauzlis [66].

*Saccades.* Saccades, which are used to track fast-moving objects, play a role in both static and dynamic balance [59]. To test saccadic movements, the same setup as for smooth-pursuit movements was utilized, but with the dot moving at a target velocity of 300 deg/s[67].

*Gaze stabilization.* Gaze stabilization is one of the most important visuomotor mechanisms influencing balance [59]. This function relies on the coordinated interaction between the visual and vestibular systems to maintain a stable visual field through micro-saccades, even during head or body movements, ensuring continuous clear vision[56]. Gaze stabilization is primarily facilitated by the vestibulo-ocular reflex (VOR), which induces a compensatory gaze shift opposite to head movement, thus contributing to both postural and visual stability[68,69]. For testing gaze stabilization, the dot remained stationary at the center of the screen while the patient moved their head to their maximum calculated range of motion in each direction. A metronome set at 180 bpm provided the pacing for these head movements, ensuring consistent timing throughout the test.

*Rapid eye-head gaze shifts.* Rapid eye-head gaze shifts involve coordinated eye and head movements, enabling swift gaze direction changes beyond the range achievable by eye movements alone [57]. These combined movements allow for broader and faster gaze adjustments. To enable simultaneous eye and head movement, the vestibulo-ocular reflex (VOR) must be voluntarily suppressed[16], a process requiring a high level of intermuscular coordination[67]. Deficiencies in VOR suppression[70]or impairments in eye-head coordination[62]can lead to postural instability. This combined eye-head movement places specific demands on motor control and involves cervical spine motion, potentially impacting balance control and oculomotor function[71]. Patients with PD, in particular, often experience difficulties with such movements[67]. To test rapid eye-head gaze shifts, the maximum range of cervical spine motion was first determined, and the midpoint gaze position was set using a laser headlamp (Concussion Lab, Toronto, Canada). This reference point was marked 0.6 m away as the target in each direction (left, right, above, and below) using two extendable Cullmann Alpha 2800 tripods (Cullmann, Fürth, Germany) and black adhesive markers (3 cm in diameter). The patients then performed simultaneous eye and head movements toward each target, paced by a metronome set at 180 bpm to ensure consistent timing.

### 2.4 Description of the intervention programs

#### 2.4.1 Outdoor Nordic walking exercise (CON)

Outdoor NW served as the CON intervention, a widely practiced training method for PD patients[72], with primary benefits observed in balance, walking ability, postural control, and coordination between upper and lower extremities. Reduced arm swing is one of the earliest PD symptoms, impacting walking dynamics, balance, and stability; the arm swing promoted by NW poles supports balance, trunk alignment, and rotation [73]. NW was conducted outdoors to reflect realistic clinical practice and everyday conditions, thereby ensuring ecological validity and clinical applicability[72,73].

NW has also demonstrated cognitive and psychological benefits, enhancing attention and focus due to exercise complexity, along with improved mood and quality of life from regular, moderate endurance training[74,75]. The NW intervention consisted of 60-minute sessions, conducted five mornings per week over four weeks and supervised by a NW-certified sports science student who was blinded to the study’s purpose.

#### 2.4.2 Treadmill and oculomotor Dual-Task exercise (ALT)

The ALT intervention was inspired by Bang and Shin [11], who compared NW on a treadmill with standard treadmill walking, finding that both interventions improved gait speed, distance, and reduced fall risk, with NW on a treadmill yielding greater benefits. This indicates that dual-tasks during walking, such as oculomotor exercises, may enhance therapy outcomes. Consequently, ALT involves the patients performing oculomotor dual-tasks while walking on a curved treadmill.

The oculomotor system significantly influences cognitive processes involved in postural balance, making it a valuable component for dual-task interventions[54]. Oculomotor changes, such as reduced fixation stability, peripheral awareness, and saccadic movements, correlate with both motor and non-motor PD symptoms[14]. Studies suggest that oculomotor training, including eye-head movements, VOR, and saccades, positively impacts static steady-state balance and may enhance dynamic balance as well[54,62]. Gaze stability exercises also reduce fall risk and postural instability, while improving dynamic visual acuity[12,68,76].

On a neurofunctional level, oculomotor exercises activate subcortical areas involved in postural control and sensorimotor circuits, enhancing functional connectivity between the peripheral nervous system and primary visual cortex[62,77]. The oculomotor system acts as a mediator between motor and cognitive functions[78]. While NW activates cortical regions such as the premotor and primary motor cortices, along with subcortical regions like the cerebellum[79,80], oculomotor dual-tasks engage brain areas especially relevant to PD, as outlined in Table S2.

In patients with PD, activity in key brain areas such as the mesencephalic locomotor region, parietal and cingulate regions, and vermis cerebelli is notably reduced[79]. Other regions implicated include the primary motor and occipital cortex, basal ganglia, and cerebellum, where visual information is processed[16,81]. Complex cognitive processes are enabled by functional connections between various brain regions; oculomotor areas interact closely with each other and with regions not primarily associated with eye movements[82]. During oculomotor training, precise coordination of eye muscles with contralateral and multisensory stimuli is required[16].

The ALT intervention also incorporates visual deprivation tasks, shown to benefit balance in PD patients[65]. Closing the eyes activates memory-related areas in the brain, aiding interoception—particularly relevant as interoception is often impaired in PD[83,84]. Brief visual deprivation can stimulate the visual system, increasing neuronal and oculomotor activity[85]. Oculomotor performance has been correlated with functional connectivity across cortical, limbic, thalamic, cerebellar, and brainstem regions, forming the neuroscientific basis for these interventions[86].

ALT sessions were held five times weekly in the morning (8–11 a.m.), with dynamic balance training on a curved treadmill combined with oculomotor dual-tasks for 20 min. Unlike NW or regular eye movements, targeted oculomotor tasks impose higher cognitive demands, justifying the shorter training duration[87]. A curved treadmill (Skillmill, Elite, Victoria, Australia) was chosen for its ability to require greater whole-body coordination and sensory integration, resembling daily-life locomotion demands[88,89]. Each session began with a five-minute warm-up, transitioning smoothly into a 20-minute high-intensity program where the patients aimed to reach and maintain maximum walking speed.

To vary muscular load, exercises were conducted in a randomized sequence using the Randomizer app (Randomness and Integrity Services Ltd., Dublin, Ireland), ensuring all nine dual-tasks were completed within 20 min. Tasks were announced by non-blinded sports science students, with repetitions counted externally to reduce cognitive load for the patients. During visual deprivation tasks, the patients closed their eyes for up to 10 s or as long as they felt safe without needing support.

### 2.5 Statistical analysis

All data are presented as mean ± standard deviation (SD). Normality of distribution for each outcome variable was assessed using the Shapiro–Wilk test. Depending on the distribution, either parametric or non–parametric procedures were applied. For normally distributed variables, paired–samples t–tests were used to compare differences between test points (T1–T2, T2–T3, and T1–T3). For non–normally distributed variables, the Wilcoxon signed–rank test was applied.

Effect sizes for pairwise comparisons were calculated as Pearson’s r, with thresholds interpreted as small (*|r|* = 0.10–0.29), moderate (*|r|* = 0.30–0.49), and large (*|r|* ≥ 0.50)[90]. Statistical significance was set at *p* < 0.05. For descriptive purposes, effect size codes are indicated in the tables as # (small), ## (moderate), and ### (large)[91].

All analyses were performed using SPSS software (version 26.0; IBM, USA). Given the pilot character of this study and the absence of a formal a priori power calculation, the current sample size reflects the number of participants who had completed both interventions at the time of analysis. As such, results should be interpreted as preliminary estimates to inform sample size calculations for future confirmatory trials.

## 3 Results

All participants completed both therapy programs as scheduled. The attendance rates during therapy sessions amounted to 95.6 % for CON (19.11 ± 1.17 sessions) and 93.9 % for ALT (18.78 ± 1.48 sessions). Attendance ranged from 17 to 20 sessions in CON and from 16 to 20 sessions in ALT. Missed sessions were primarily due to illness or personal scheduling conflicts.

### 3.1 Anthropometric measurements, quality of life and motor examinations

As shown in Table 2, after CON (T2), small improvements in anthropometric parameters were observed. BMI decreased from 25.90 ± 2.84 to 25.48 ± 2.86 (*r* = 0.35) and body fat percentage from 23.76 ± 5.61 to 23.17 ± 5.60 (*r* = 0.37). While the Hoehn and Yahr stage remained unchanged, MDS‑UPDRS total score, improved from 62.44 ± 13.13 to 58.33 ± 13.34 (*r* = 0.33). Quality of life improved, with PDQ‑39 total score decreasing from 30.07 ± 2.72 to 22.59 ± 2.18 (*r* = 0.33). The largest changes occurred in mobility (*r* = 0.35), activities of daily living (*r* = 0.39) and cognition (*r* = 0.33), while emotional well‑being and stigma improved only modestly (*r* ≤ 0.30).

**Table 2:** Results of anthropometric measurements, quality of life, and motor assessments.

Test procedure		*p* (T1–T3)	*r* (T1–T3)	*p* (T1–T2)	*r* (T1–T2)	*p* (T2–T3)	*r* (T2–T3)
Body height [cm]		−	−	−	−	−	−
BMI		0.001*	0.65###	0.08	0.35##	0.18	0.30##
Body fat		0.001*	0.63###	0.06	0.37##	0.29	0.26#
Hoehn-and-Yahr-Scale		−	−	−	−	−	−
PDQ-39 total score		0.001*	0.66###	0.1	0.33##	0.1	0.33##
	1. Mobility	0.001*	0.65###	0.08	0.35##	0.18	0.30##

	2. Activities of daily living	0.001*	0.61###	0.04*	0.39##	0.47	0.22#
	3. Emotional well-being	0.001*	0.65###	0.18	0.30##	0.08	0.35##
	4. Stigma	0.001*	0.65###	0.18	0.30##	0.08	0.35##
	5. Social support	0.17	0.06###	0.40	0.28#	0.73	−0.12
	6. Cognitions	0.001*	0.61###	0.10	0.33##	0.27	0.30##
	7. Communication	0.73	0.07	0.34	−0.32	0.74	0.11
	8. Pain	0.001*	0.61###	0.23	0.07	0.1	0.33##
MDS-UPDRS Total		0.001*	0.66###	0.1	0.33##	0.1	0.33##
	Part 1	0.001*	0.66###	0.1	0.33##	0.1	0.33##
	Part 2	0.001*	0.66###	0.1	0.33##	0.1	0.33##
	Part 3	0.001*	0.66###	0.1	0.33##	0.1	0.33##
	Part 4	−	−	−	−	−	−

*Note.* T1-T3: Testing 1–3; BMI: Body Mass Index; PDQ-39: Parkinson’s Disease Questionnaire; MDS-UPDRS: Movement Disorder Society-sponsored revision of the Unified Parkinson’s Disease Rating Scale; * = statistical significance; #, ##, ### = small, moderate, and large effects, respectively.

After ALT (T3), anthropometric measures showed continued small but non-significant improvements. BMI decreased from 25.48 ± 2.86 to 25.30 ± 2.84 (*r* = 0.30) and body fat from 23.17 ± 5.60 to 22.91 ± 5.51 (*r* = 0.26). The MDS‑UPDRS total score further decreased from 58.33 ± 13.34 to 53.78 ± 14.49 (*r* = 0.33), primarily driven by Part 3 (motor examination: *r* = 0.33).

Distinct from CON, ALT led to improvements in affective quality‑of‑life domains. Emotional well‑being improved from 18.98 ± 5.15 to 13.43 ± 4.56 (*r* = 0.35) and stigma from 46.53 ± 17.98 to 33.33 ± 13.62 (*r* = 0.35). Mobility (*r* = 0.30), activities of daily living (*r* = 0.22) and cognition (*r* = 0.30) also continued to improve, though less markedly than in the CON phase. Communication and social support showed no meaningful improvement, with effect sizes close to zero and in some cases slightly negative, indicating small fluctuations without clinical relevance.

In summary, both interventions produced beneficial changes in anthropometric parameters, motor performance, and quality of life. CON yielded broader initial gains in physical domains, while ALT provided additional and clinically relevant improvements particularly in non‑motor and affective domains.

### 3.2 Proactive and dynamic steady-state balance performance

Table 3 summarises the outcomes of the proactive and dynamic steady‑state balance assessments. After CON (T2), small performance gains were observed (*r* = 0.19–0.41). The YBT‑LQ composite score improved from 102.91 ± 5.31 to 103.60 ± 5.32 for the left leg (*r* = 0.19) and from 102.81 ± 7.22 to 103.48 ± 6.80 for the right leg (*r* = 0.26). Gains were evident across most reach directions, with the largest effect in the posteromedial right direction (*r* = 0.41). 10MWT performance slightly decreased from 108.67 ± 5.47 to 106.10 ± 4.41  m/s (*r* = 0.15), while MiniBESTest scores remained stable (*r* = 0.02).

**Table 3:** Results of proactive and dynamic steady-state balance performance.

Test procedure	*p* (T1–T3)	*p* (T1–T2)	*r* (T1–T2)	*p* (T2–T3)	*r* (T2–T3)
YBT-LQ score L	0.001*	0.72	0.19#	0.029*	0.41##
YBT-LQ score R	0.001*	0.3	0.26#	0.045*	0.39##
Anterior L	0.001*	0.23	0.28#	0.1	0.33##
Anterior R	0.002*	1	0.07	0.01*	0.46##
postero-medial L	0.001*	0.1	0.33##	0.1	0.33##
postero-medial R	0.001*	0.03	0.41##	0.72	0.18#
postero-lateral L	0.001*	0.18	0.30##	0.07	0.35##
postero-lateral R	0.001*	0.72	0.19#	0.3	0.41##
10MWT [m/s]	0.007*	1	0.15#	0.02*	0.43##
MiniBESTest	0.001*	1	0.02	0.01*	0.46##

*Note.* T1-T3: Testing 1–3; YBT-LQ: Y-Balance Test Lower Quarter composite score; MiniBESTest: Mini Balance Evaluation Systems Test; * = statistical significance; #, ##, ### = small, moderate, and large effects, respectively.

After ALT (T3), further improvements in balance performance were evident, some reaching statistical significance (*p* < 0.05, *r* = 0.18–0.46). The YBT‑LQ composite score increased from 103.60 ± 5.32 to 106.21 ± 5.00 for the left leg (*p* = 0.029, *r* = 0.41) and from 103.48 ± 6.80 to 105.68 ± 6.41 for the right leg (*p* = 0.045, *r* = 0.39). The anterior right reach showed a notable gain from 71.30 ± 2.41 to 69.98 ± 2.72 (*p* = 0.010, *r* = 0.46), while the MiniBESTest score increased from 102.37 ± 3.07 to 104.82 ± 3.23 (*p* = 0.010, *r* = 0.46).

In summary, CON produced modest balance improvements, with the largest effects in specific YBT‑LQ reach directions, while ALT elicited additional and in some cases statistically significant gains in both composite YBT‑LQ scores and clinical balance measures, suggesting enhanced dynamic balance control following the treadmill‑oculomotor intervention.

### 3.3 Static steady-state balance performance

**Fig. 2:**
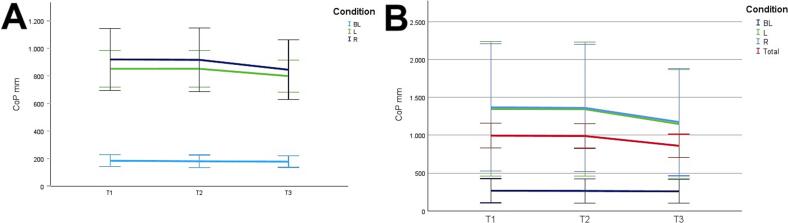

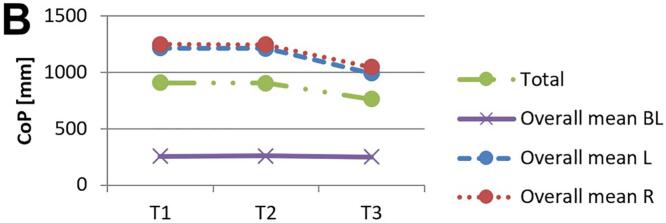
A-B. Results of static steady-state balance performance without dual-tasks (A) and overall mean value with dual-tasks (B). *Note.* BL: bilateral; L: left; R: right; CoP: Center of Pressure.

**Fig. 3:**
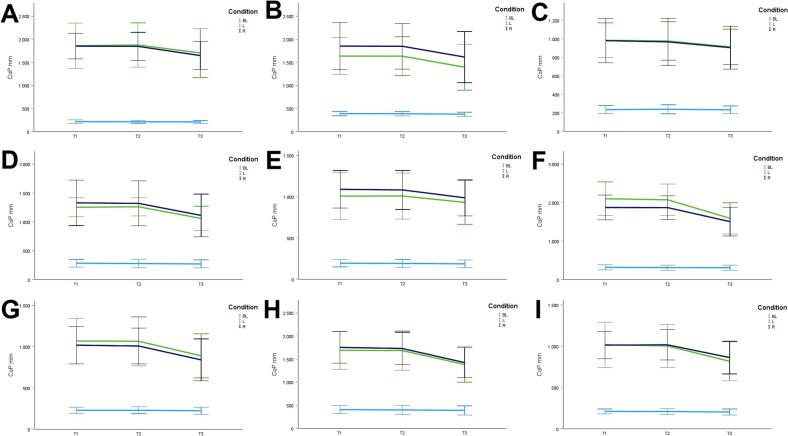

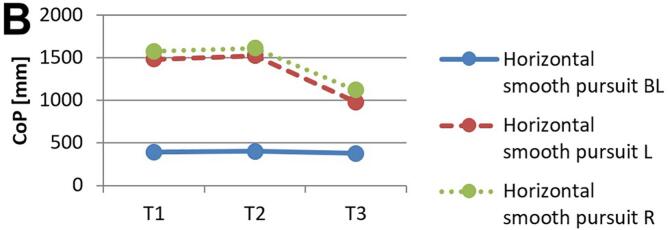

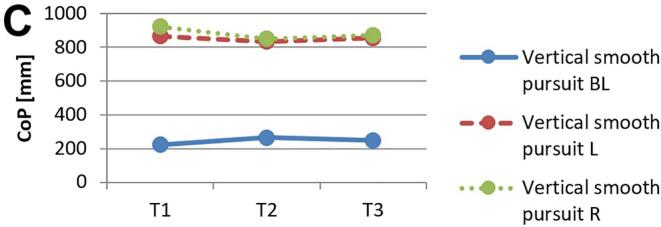

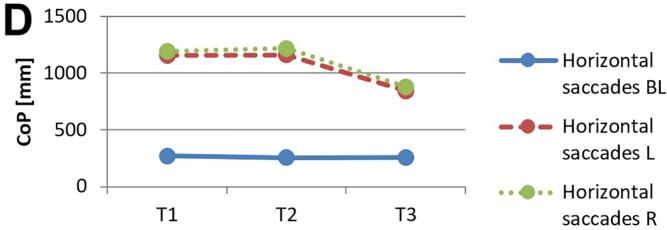

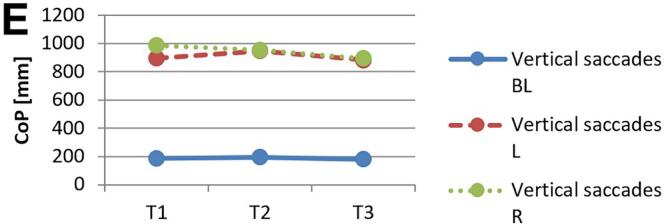

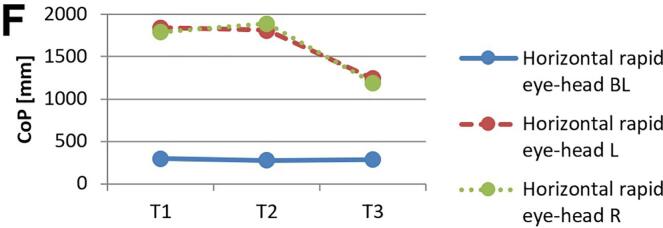

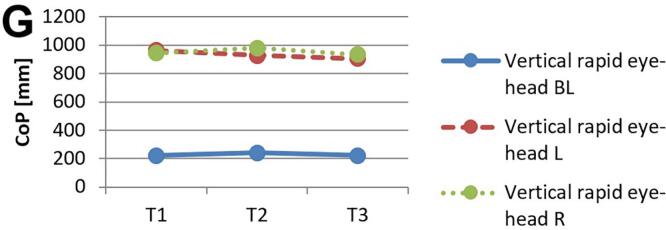

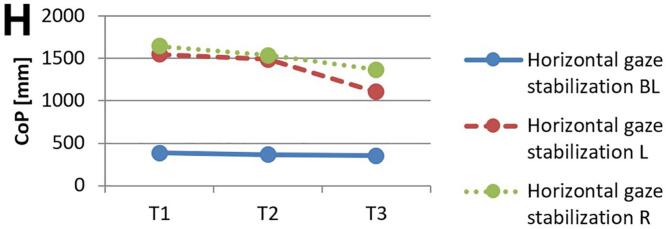

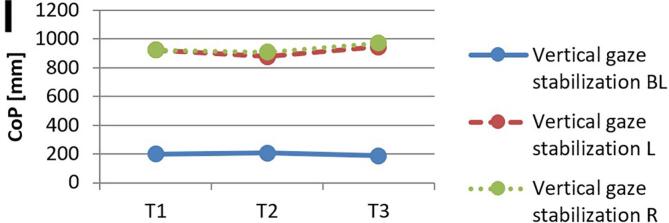
A-I. Results of static steady-state balance performance with eyes closed (A), horizontal smooth pursuit eye movements (B), vertical smooth pursuit eye movements (C), horizontal saccades (D), vertical saccades (E), horizontal rapid eye-head gaze shifts (F), vertical rapid eye-head gaze shifts (G), horizontal gaze stabilization (H), vertical gaze stabilization (I). *Note.* BL: bilateral; L: left; R: right; CoP: Center of Pressure.

Fig. 2 and 3 provide an overview of static steady–state balance performance with and without oculomotor dual–tasks, showing CoP values across the three test times.

During CON (T1–T2), balance performance remained largely stable across all conditions, with all p > 0.05 and small effect sizes (*r* = 0.05–0.21). In the no–dual–task condition (Fig. 2A), changes were negligible, and the overall mean CoP across all dual–task conditions (Fig. 2B) showed no significant change from T1 to T2 (*p* = 0.75, *r* = 0.05). The largest change during CON was observed in horizontal gaze stabilization on the right leg (Fig. 3H; *r* = 0.34), although this did not reach statistical significance (*p* = 0.12).

In contrast, during ALT (T2–T3), several improvements reached statistical significance and demonstrated moderate–to–large effect sizes. The overall CoP across all dual–task conditions (Fig. 2B) was significantly reduced (*p* = 0.04, *r* = 0.53). The largest improvements occurred in horizontal smooth pursuit (Fig. 3B; left leg: *p* = 0.02, *r* = 0.55; right leg: *p* = 0.01, *r* = 0.59), horizontal saccades (Fig. 3D; right leg: *p* = 0.02, *r* = 0.56), and horizontal rapid eye–head gaze shifts (Fig. 3F; right leg: *p* = 0.01, *r* = 0.59). Moderate effects were also observed in vertical rapid gaze shifts (Fig. 3G; *r* = 0.44) and vertical gaze stabilization (Fig. 3I; *r* = 0.43), although these were not statistically significant (*p* > 0.05).

In summary, CON predominantly maintained static balance performance, with only minor fluctuations, whereas ALT produced significant and moderate–to–large improvements, particularly in horizontal oculomotor dual–task conditions, suggesting enhanced postural control following the treadmill–oculomotor intervention.

## 4 Discussion

### 4.1 Proactive and dynamic steady-state balance performance

The improvements in gait speed and dynamic balance observed after the CON intervention align with the known effects of NW in the general population[75].

Similarly, Bang and Shin [11] reported that both NW and treadmill interventions enhanced balance, with NW showing greater improvements in dynamic balance, likely due to the added motor complexity from coordinating upper and lower limbs. In contrast, the present findings indicate that the treadmill‑oculomotor intervention elicited larger gains in dynamic balance measures than NW, suggesting that the integration of targeted oculomotor tasks may provide an additional stimulus beyond the motor complexity inherent to NW.

Recent systematic reviews focusing on PD patients suggest that NW offers beneficial effects compared to other exercise interventions, with large effects on gait speed but only small effects on balance[72,92]. In the present study, CON resulted in only modest changes in proactive and dynamic balance parameters, consistent with these reports. Similarly, Bang and Shin [11] found that while both NW and treadmill interventions enhanced balance, NW led to significantly higher dynamic balance improvements, likely due to the added motor complexity from coordinating upper and lower limbs.

In contrast, after ALT, additional and in some cases statistically significant improvements were observed in gait speed and in the third motor part of the MDS–UPDRS, alongside clear gains in YBT–LQ composite scores for both legs (*r* = 0.39–0.41) and in the Mini–BESTest (*r* = 0.46). These findings support previous reports on the benefits of oculomotor exercises for dynamic balance[62,77]and extend them by demonstrating their effectiveness in a younger PD cohort.

While the Mini–BESTest score increased only slightly after CON, the improvement following ALT exceeded the threshold for a clinically meaningful change, highlighting the added value of incorporating oculomotor tasks into treadmill training. Similarly, although the YBT–LQ performance of the right leg declined after CON, both legs improved after ALT, with gains evident across multiple reach directions and the largest effects in the anterior right reach.

Oculomotor dual‑task conditions likely enhance sensory integration and coordination between the visual and motor systems, as seen in prior research on patients with acquired brain injuries[17,93]. The increased cognitive load imposed by these tasks activates the CNS and promotes neuroplasticity[94]. Integrating visual and motor challenges during treadmill walking may strengthen functional connections between these systems, thereby improving coordination and control in unilateral movements[68,77]. The present results, particularly the significant reductions in CoP under specific visuo‑motor conditions, reinforce this interpretation and suggest that the dynamic balance gains following ALT are underpinned by enhanced visual‑motor integration.

### 4.2 Static steady-state balance performance

In evaluating static steady–state balance performance, results indicate minimal impact from CON, while ALT produced significant improvements, as shown by a marked reduction in overall mean CoP (*r* = 0.53). ALT demonstrated greater enhancements in eyes–closed conditions, unilateral tasks, and horizontal eye–movement conditions compared to CON. The strongest effects were observed for horizontal smooth pursuit (*r* = 0.55–0.59), horizontal saccades (*r* = 0.56), and horizontal rapid eye–head gaze shifts (*r* = 0.59). In contrast, CON induced only small, non–significant changes across these measures. Participants generally performed worse on horizontal than on vertical tasks, which may relate to vestibular sensitivity differences; specifically, the utricle detects vertical and the saccule horizontal acceleration, suggesting individual variability in response to movement direction[15,95]. Oculomotor dual–task training may enhance control and integration of horizontal eye–movement information, potentially linked to increased activation in the frontal eye field, which is associated with balance and eye–movement control[96].

While ALT improved overall balance performance, primarily in unilateral static steady–state tasks, the data highlight ALT’s advantage over CON rather than effects of specific oculomotor conditions alone. Although Rodrigues et al. [55] found that horizontal saccades reduced sway and smooth pursuit increased it, the present results show greater improvements in unilateral than bilateral stance, suggesting that visuo–spatial load during treadmill walking preferentially benefits unilateral balance. According to Hadian et al. [77], oculomotor exercises improve coordination between head, body, and pelvis during walking, supporting neuromuscular reorganization through neuroplasticity. The observed improvement in single–leg stance under dual–task conditions, compared to bilateral stance, can be explained by the increased neuromuscular and cognitive demands associated with single–leg postural control. Single–leg stance requires greater activation of cortical and subcortical networks, particularly within the sensorimotor cortex and cerebellum, to maintain balance with limited support[97]. Dual–task training enhances the brain’s capacity for divided attention and resource allocation across motor and cognitive tasks, which is particularly beneficial for tasks that challenge balance and coordination[98]. In contrast, bilateral stance relies more on automatic postural mechanisms with less demand on higher–level cortical processing, resulting in a lower sensitivity to dual–task improvements[99]. Thus, the specificity of dual–task training more profoundly benefits complex, unilateral balance tasks.

Equal average improvements in both legs suggest that adaptations in the vestibular nuclei and cerebellum may contribute to improved static balance. Oculomotor exercises positively affect neural circuits and CNS plasticity, which is particularly relevant in early–stage PD where neuroplasticity significantly influences disease progression[100,101]. Although this study did not measure molecular–level changes in brain connectivity, previous research suggests that neuronal adaptation may underlie these improvements[82,83].

### 4.3 Quality of life, non-motor symptoms and patient’s perspective

In terms of non–motor symptoms, the CON intervention positively impacted quality of life, particularly in the domains of daily activities and physical discomfort, consistent with findings by Tschentscher et al. [75]. Improvements in physical discomfort, such as reduced muscle cramps, joint pain, and temperature sensitivity, may be attributed to increased overall physical activity rather than to NW specifically[102].

In contrast, ALT elicited distinct and clinically relevant improvements in the affective domains of emotional well–being and stigma (*r* = 0.35 for both), which were not observed following CON. These changes are likely attributable to the oculomotor dual–tasks, which are known to enhance functional connectivity between the visual and limbic systems[103]. This supports the hypothesis that improving functional connectivity through ALT can lead to better non–motor outcomes. Eye–movement tasks also engage brain regions such as the prefrontal cortex and hippocampus, which are involved in emotional processing and regulation[16]. Psychological benefits reported in neurological conditions such as multiple sclerosis after visual–motor training[12]further support this interpretation, although this relationship has been explored only to a limited extent in PD.

From the participants’ perspective, both interventions were perceived as beneficial; however, ALT was particularly associated with increased confidence in cognitively demanding situations. Several participants reported a subjective reduction in symptom exacerbation during mentally challenging tasks and described feeling more in control during professional and social interactions. In contrast, CON was favored for its emotional and sensory qualities, especially the outdoor setting. Participants consistently highlighted the mood-enhancing effect of daylight and the stimulating impact of the changing environment, which were perceived as essential contributors to overall well-being. For some, these factors were considered more important than the shorter duration and indoor feasibility of the treadmill-based training, emphasizing the relevance of natural environments in shaping perceived therapeutic benefit.

### 4.4 Limitations

A primary limitation of this study is the small sample size, which results in low statistical power and limits the ability to detect smaller but clinically relevant effects. In addition, the absence of a control group increases the risk of psychological bias, such as confirmation bias, which may influence both participants’ expectations and the interpretation of outcomes[104]. Although the sequential within–subjects case series design was chosen due to the novelty and complexity of the intervention protocol, this approach limits the generalizability of the findings and reduces the ability to draw strong causal inferences. As a pilot study, the primary aim was to establish feasibility, assess participant compliance, and collect preliminary effect estimates to inform the design of future randomized controlled trials. Such trials should include larger and more diverse samples and an appropriate control condition to allow for broader interpretation of results.

Another methodological limitation is the fixed order of the interventions, with NW preceding the oculomotor dual–task training. This sequencing introduces the potential for order effects and carry–over bias. While this sequence was intentionally chosen to reflect real–world therapy progression and feasibility, it limits the ability to isolate effects specific to each intervention. Additionally, the absence of a consolidation or wash–out phase between interventions poses limitations. Potential carry–over effects from CON to ALT could have been minimized with a wash–out phase after the first intervention[105]. However, this was not feasible due to time constraints and ethical considerations, as a therapy–free period would have been impractical. Such a phase might have been beneficial, as vestibular adaptations are not typically reversible in the short term[106]. The carry–over effects from NW may have influenced subsequent measurements, as sustained activity–related improvements have been reported even months after intervention[107]. While this approach prioritized clinical feasibility and participant safety, it limits the ability to fully disentangle the effects of each intervention. Future studies should incorporate a counterbalanced crossover design with appropriate wash–out periods to address these issues.

A further limitation is the absence of follow–up data to assess the sustainability of intervention effects. Rehabilitation outcomes, particularly in chronic neurodegenerative conditions such as PD, may evolve over time, and long–term follow–up is critical to determine whether observed gains are maintained. Additionally, the limited duration of four weeks per intervention restricts the assessment of long–term effectiveness. Future trials should consider intervention periods of 8–12 weeks and follow–ups at three and six months to capture lasting benefits or potential regression across motor and non–motor outcomes.

There may also have been learning or sequencing effects due to the similarity of NW with other walking and exercise tasks. Although counterbalancing the interventions could have mitigated these effects[108], NW was intentionally chosen as a comparison to explore a time–efficient and widely applicable alternative.

Moreover, conducting NW outdoors rather than on a treadmill might have introduced a confounding factor. However, Lu et al. [109] reported that differences between treadmill and overground walking in PD are minor, suggesting a limited influence of this methodological choice on the present results. Nonetheless, subjective feedback from participants indicated that the outdoor environment of the NW intervention was perceived as a meaningful component of the therapeutic experience. This highlights that contextual factors, while not necessarily influencing motor outcomes, may play an important role in perceived effectiveness and adherence.

Lastly, while five trials are generally recommended for accurate static steady–state balance measurement[110], only three were performed to limit participant burden. Given the demanding oculomotor testing protocol, a full set of five trials could have increased fatigue, potentially confounding the results due to exhaustion.

## 5 Conclusions

This pilot study provides preliminary evidence that treadmill–based training combined with oculomotor dual–tasks can elicit meaningful improvements in both proactive and static balance performance in people with PD. Gains were particularly evident in unilateral tasks and during horizontal eye–movement conditions, which are known to challenge sensory–motor integration. In addition to motor–related improvements, the oculomotor protocol uniquely enhanced non–motor domains such as emotional well–being and stigma, outcomes that were not observed following CON. These findings suggest that targeted visual–motor integration training may complement established exercise approaches in PD rehabilitation.

While ALT was followed by more pronounced effects on specific balance parameters, the fixed intervention order limits causal interpretation. Nonetheless, the shorter duration and indoor feasibility offer practical advantages over CON.

Oculomotor dual–tasks remain an underexplored avenue in PD therapy. Their potential to stimulate neural circuits involved in visual–motor coordination and to promote neuroplasticity underscores the need for their integration into broader rehabilitation frameworks. Future research should confirm these findings in larger and more diverse PD cohorts, include appropriate control conditions, and examine the persistence of benefits over time. Randomized and counterbalanced designs with sufficient wash–out periods are recommended to better isolate intervention–specific effects and clarify whether the observed greater responsiveness to horizontal compared to vertical oculomotor conditions can be consistently replicated.

## 6 Consent to participate

Written informed consent was obtained from all participants.

## 7 Consent for publication

Written informed consent for publication was obtained from all participants.

## Data availability statement

The original contributions presented in the study are included in the article/supplementary material, further inquiries can be directed to the corresponding author/s.

## CRediT authorship contribution statement

**Marc Niering:** Writing – review & editing, Writing – original draft, Methodology, Formal analysis, Conceptualization. **Corinna Wirth:** Writing – original draft, Methodology, Conceptualization. **Rainer Beurskens:** Writing – review & editing, Formal analysis. **Elisa Ueding:** Writing – review & editing, Formal analysis. **Tim Fischer:** Methodology. **Johanna Seifert:** Writing – review & editing, Formal analysis.

## Ethics approval and consent to participate

Approval for the study protocol was obtained from the Human Ethics Committee at the Nordic Science Institute of Biomechanics and Neurosciences, Hannover, Germany, HEC-IBN-2024–10.

## Declaration of competing interest

The authors declare that they have no known competing financial interests or personal relationships that could have appeared to influence the work reported in this paper. Furthermore, the authors received no funding for this work.

## References

[1] Freidle M., Johansson H., Ekman U., Lebedev A.V., Schalling E., Thompson W.H., Svenningsson P., Lövdén M., Abney A., Albrecht F., Steurer H., Leavy B., Holmin S., Hagströmer M., Franzén E. (2022). Behavioural and neuroplastic effects of a double-blind randomised controlled balance exercise trial in people with Parkinson’s disease. Npj Parkinson’s Disease.

[2] Hartkamp N. (2022). Morbus Parkinson: Somatik und Psychosomatik. Ärztliche Psychotherapie.

[3] Balestrino R., Schapira A.H.V. (2020). Parkinson disease. Eur. J. Neurol..

[4] Armstrong M.J., Okun M.S. (2020). Diagnosis and treatment of Parkinson disease: a review. JAMA.

[5] Dorsey E.R., Elbaz A., Nichols E., Abbasi N., Abd-Allah F., Abdelalim A., Adsuar J.C., Ansha M.G., Brayne C., Choi J.-Y.-J., Collado-Mateo D., Dahodwala N., Do H.P., Edessa D., Endres M., Fereshtehnejad S.-M., Foreman K.J., Gankpe F.G., Gupta R., Murray C.J.L. (2018). Global, regional, and national burden of Parkinson’s disease, 1990–2016: a systematic analysis for the Global Burden of Disease Study 2016. The Lancet Neurology.

[6] Bhalsing K.S., Abbas M.M., Tan L.C.S. (2018). Role of Physical activity in Parkinson’s Disease. Ann. Indian Acad. Neurol..

[7] Bloem B.R., Okun M.S., Klein C. (2021). Parkinson’s disease. Lancet.

[8] Virameteekul S., Phokaewvarangkul O., Bhidayasiri R. (2021). Profiling the most elderly parkinson’s disease patients: does age or disease duration matter?. PLoS One.

[9] Albrecht F., Pereira J.B., Mijalkov M., Freidle M., Johansson H., Ekman U., Westman E., Franzén E. (2021). Effects of a Highly Challenging Balance Training Program on Motor Function and Brain Structure in Parkinson’s Disease. Journal of Parkinson’s Disease.

[10] Silva-Batista C., Corcos D.M., Roschel H., Kanegusuku H., Gobbi L.T.B., Piemonte M.E.P., Mattos E.C.T., De Mello M.T., Forjaz C.L.M., Tricoli V., Ugrinowitsch C. (2016). Resistance Training with Instability for patients with Parkinson’s Disease. Med. Sci. Sports Exerc..

[11] Bang D.-H., Shin W.-S. (2017). Effects of an intensive Nordic walking intervention on the balance function and walking ability of individuals with Parkinson’s disease: a randomized controlled pilot trial. Aging Clin. Exp. Res..

[12] Hebert J.R., Corboy J.R., Vollmer T., Forster J.E., Schenkman M. (2018). Efficacy of Balance and Eye-Movement Exercises for Persons with Multiple Sclerosis (BEEMS). Neurology.

[13] Nilsson M.H., Patel M., Rehncrona S., Magnusson M., Fransson P.-A. (2013). Subthalamic deep brain stimulation improves smooth pursuit and saccade performance in patients with Parkinson’s disease. J. Neuroeng. Rehabil..

[14] Zhang Y., Yan A., Liu B., Wan Y., Zhao Y., Liu Y., Tan J., Song L., Gu Y., Liu Z. (2018). Oculomotor Performances are Associated with Motor and Non-motor Symptoms in Parkinson’s Disease. Front. Neurol..

[15] Heermann S. (2017). Neuroanatomie des okulomotorischen Systems. Klinische Monatsblätter Für Augenheilkunde.

[16] Trepel M. (2022).

[17] Niering M., Seifert J. (2024). The effects of visual skills training on cognitive and executive functions in stroke patients: a systematic review with meta-analysis. J. Neuroeng. Rehabil..

[18] Beurskens R., Haeger M., Kliegl R., Roecker K., Granacher U. (2016). Postural Control in Dual-Task Situations: does Whole-Body Fatigue Matter?. PLoS One.

[19] Waldthaler J., Tsitsi P., Svenningsson P. (2019). Vertical saccades and antisaccades: Complementary markers for motor and cognitive impairment in Parkinson’s disease. Npj Parkinson’s Disease.

[20] Keller M., Roth R., Achermann S., Faude O. (2022). Learning a new balance task: the influence of prior motor practice on training adaptations. Eur. J. Sport Sci..

[21] Horne J.A., Ostberg O. (1976). A self-assessment questionnaire to determine morningness-eveningness in human circadian rhythms. Int. J. Chronobiol..

[22] Soares N.M., Pereira G.M., Altmann V., de Almeida R.M.M., Rieder C.R.M. (2019). Cortisol levels, motor, cognitive and behavioral symptoms in Parkinson’s disease: a systematic review. J. Neural Transm..

[23] Roy S., Field G.D. (2019). Dopaminergic modulation of retinal processing from starlight to sunlight. J. Pharmacol. Sci..

[24] Lambert G., Reid C., Kaye D., Jennings G., Esler M. (2002). Effect of sunlight and season on serotonin turnover in the brain. Lancet.

[25] Yang Y.-R., Cheng S.-J., Lee Y.-J., Liu Y.-C., Wang R.-Y. (2019). Cognitive and motor dual task gait training exerted specific training effects on dual task gait performance in individuals with Parkinson’s disease: a randomized controlled pilot study. PLoS One.

[26] Gribble P.A., Tucker W.S., White P.A. (2007). Time-of-day influences on static and dynamic postural control. J. Athl. Train..

[27] Kara M., Patlar S., Stoffregen T.A., Erkmen N. (2018). Effect of caffeine on standing balance during perceptual-cognitive tasks. Malaysian Journal of Movement, Health & Exercise.

[28] Ołpińska-Lischka M., Kujawa K., Maciaszek J. (2021). Differences in the effect of sleep Deprivation on the Postural Stability among men and Women. Int. J. Environ. Res. Public Health.

[29] Berardi A., Regoli E., Tofani M., Valente D., Fabbrini G., Fabbrini A., Ruggieri M., Panuccio F., Galeoto G. (2021). Tools to assess the quality of life in patients with Parkinson’s disease: a systematic review. Expert Rev. Pharmacoecon. Outcomes Res..

[30] Jenkinson C., Fitzpatrick R., Peto V., Greenhall R., Hyman N. (1997). The Parkinson’s Disease Questionnaire (PDQ-39): Development and validation of a Parkinson’s disease summary index score. Age Ageing.

[31] Schädler S. (2011). Parkinson’s Disease Questionnaire (PDQ-39)—Das Leben zu Hause im Blick. Physiopraxis.

[32] Goetz C.G., Poewe W., Rascol O., Sampaio C., Stebbins G.T., Counsell C., Giladi N., Holloway R.G., Moore C.G., Wenning G.K., Yahr M.D., Seidl L. (2004). *Movement* Disorder Society Task Force report on the Hoehn and Yahr staging scale: Status and recommendations the *Movement* Disorder Society Task Force on rating scales for Parkinson’s disease. Mov. Disord..

[33] Martinez-Martin, P., Skorvanek, M., Rojo-Abuin, J. M., Gregova, Z., Stebbins, Glenn. T., Goetz, C. G., & members of the QUALPD Study Group (2018). Validation study of the hoehn and yahr scale included in the MDS-UPDRS: Validation of the Hoehn and Yahr Scale. Mov. Disord..

[34] Goetz C.G., Tilley B.C., Shaftman S.R., Stebbins G.T., Fahn S., Martinez-Martin P., Poewe W., Sampaio C., Stern M.B., Dodel R., Dubois B., Holloway R., Jankovic J., Kulisevsky J., Lang A.E., Lees A., Leurgans S., LeWitt P.A., Nyenhuis D., LaPelle N. (2008). Movement Disorder Society-sponsored revision of the Unified Parkinson’s Disease Rating Scale (MDS-UPDRS): Scale presentation and clinimetric testing results: MDS-UPDRS: Clinimetric Assessment. Mov. Disord..

[35] Regnault A., Boroojerdi B., Meunier J., Bani M., Morel T., Cano S. (2019). Does the MDS-UPDRS provide the precision to assess progression in early Parkinson’s disease? Learnings from the Parkinson’s progression marker initiative cohort. J. Neurol..

[36] Terao Y., Fukuda H., Yugeta A., Hikosaka O., Nomura Y., Segawa M., Hanajima R., Tsuji S., Ugawa Y. (2011). Initiation and inhibitory control of saccades with the progression of Parkinson’s disease – changes in three major drives converging on the superior colliculus. Neuropsychologia.

[37] Leddy A.L., Crowner B.E., Earhart G.M. (2011). Utility of the Mini-BESTest, BESTest, and BESTest Sections for Balance Assessments in individuals with Parkinson Disease. J. Neurol. Phys. Ther..

[38] Franchignoni F., Horak F., Godi M., Nardone A., Giordano A. (2010). Using psychometric techniques to improve the Balance Evaluation Systems Test: the mini-BESTest. J. Rehabil. Med..

[39] Jagger K., Frazier A., Aron A., Harper B. (2020). Scoring performance variations between the Y-Balance Test, a modified Y-Balance Test, and the modified Star Excursion Balance Test. Int. J. Sports Phys. Ther..

[40] Plisky P., Schwartkopf-Phifer K., Huebner B., Garner M.B., Bullock G. (2021). Systematic Review and Meta-Analysis of the Y-Balance Test lower Quarter: Reliability, Discriminant Validity, and Predictive Validity. Int. J. Sports Phys. Ther..

[41] Hébert-Losier K. (2017). Clinical Implications of Hand Position and lower Limb Length Measurement Method on Y-Balance Test Scores and Interpretations. J. Athl. Train..

[42] Powden C.J., Dodds T.K., Gabriel E.H. (2019). THE RELIABILITY OF THE STAR EXCURSION BALANCE TEST AND LOWER QUARTER Y-BALANCE TEST IN HEALTHY ADULTS: a SYSTEMATIC REVIEW. Int. J. Sports Phys. Ther..

[43] Plisky P., Gorman P., Butler R., Kiesel K., Underwood F., Elkins B. (2009). The reliability of an instrumented device for measuring components of the star excursion balance test. North American Journal of Sports Physical Therapy: NAJSPT.

[44] Combs S.A., Diehl M.D., Filip J., Long E. (2014). Short-distance walking speed tests in people with Parkinson disease: Reliability, responsiveness, and validity. Gait Posture.

[45] Lindholm B., Nilsson M.H., Hansson O., Hagell P. (2018). The clinical significance of 10-m walk test standardizations in Parkinson’s disease. J. Neurol..

[46] Paillard T., Noé F. (2015). Techniques and Methods for Testing the Postural Function in healthy and Pathological Subjects. Biomed Res. Int..

[47] Harro C.C., Marquis A., Piper N., Burdis C. (2016). Reliability and Validity of Force Platform measures of Balance Impairment in individuals with Parkinson Disease. Phys. Ther..

[48] Thewlis D., Hillier S., Hobbs S.J., Richards J. (2014). Preoperative asymmetry in load distribution during quite stance persist following total knee arthroplasty. Knee Surg. Sports Traumatol. Arthrosc..

[49] Quijoux F., Nicolaï A., Chairi I., Bargiotas I., Ricard D., Yelnik A., Oudre L., Bertin-Hugault F., Vidal P., Vayatis N., Buffat S., Audiffren J. (2021). A review of center of pressure (COP) variables to quantify standing balance in elderly people: Algorithms and open‐access code*. Physiol. Rep..

[50] Barbieri F.A., Carpenter M., Beretta V.S., Orcioli-Silva D., Simieli L., Vitório R., Gobbi L.T.B. (2019). Postural control, falls and Parkinson’s disease: are fallers more asymmetric than non-fallers?. Hum. Mov. Sci..

[51] Almeida I.A.D., Terra M.B., Oliveira M.R.D., Silva Júnior R.A.D., Ferraz H.B., Santos S.M.S. (2016). Comparing postural balance among older adults and Parkinson’s disease patients. Motriz: Revista De Educação Física.

[52] Nemanich S.T., Earhart G.M. (2016). Freezing of gait is associated with increased saccade latency and variability in Parkinson’s disease. Clin. Neurophysiol..

[53] Takakusaki K. (2017). Functional Neuroanatomy for Posture and Gait Control. Journal of Movement Disorders.

[54] Hoffmann M.A., Pieczykolan A., Koch I., Huestegge L. (2019). Motor sources of dual-task interference: evidence for effector-based prioritization in dual-task control. J. Exp. Psychol. Hum. Percept. Perform..

[55] Rodrigues S.T., Polastri P.F., Carvalho J.C., Barela J.A., Moraes R., Barbieri F.A. (2015). Saccadic and smooth pursuit eye movements attenuate postural sway similarly. Neurosci. Lett..

[56] Lafleur D., Lajoie Y. (2023). The impact of eye movement on postural control depends on the type of oculomotor behavior and the visual task. Gait Posture.

[57] Laurens J., Awai L., Bockisch C.J., Hegemann S., van Hedel H.J.A., Dietz V., Straumann D. (2010). Visual contribution to postural stability: Interaction between target fixation or tracking and static or dynamic large-field stimulus. Gait Posture.

[58] Thomas, N. M., Bampouras, T. M., Donovan, T., & Dewhurst, S. (2016). Eye Movements Affect Postural Control in Young and Older Females. *Frontiers in Aging Neuroscience*, *8*. https://doi.org/10.3389/fnagi.2016.00216. 10.3389/fnagi.2016.00216 PMC5025428 27695412

[59] Kim S.-Y., Moon B.-Y., Cho H.G. (2016). Smooth-pursuit eye movements without head movement disrupt the static body balance. J. Phys. Ther. Sci..

[60] Munoz M.J., Reilly J.L., Pal G.D., Verhagen Metman L., Rivera Y.M., Drane Q.H., Corcos D.M., David F.J., Goelz L.C. (2022). Medication adversely impacts visually-guided eye movements in Parkinson’s disease. Clin. Neurophysiol..

[61] Wark H.A.C., Garell P.C., Walker A.L., Basso M.A. (2008). A case report on fixation instability in Parkinson’s disease with bilateral deep brain stimulation implants. J. Neurol. Neurosurg. Psychiatry.

[62] Abasi A., Hoseinabadi R., Raji P., Friedman J.H., Hadian M.-R. (2022). Evaluating Oculomotor Tests before and after Vestibular Rehabilitation in patients with Parkinson’s Disease: a pilot Pre-Post Study. Parkinson’s Disease.

[63] Halmágyi G.M., Curthoys I.S. (2021). Vestibular contributions to the Romberg test: Testing semicircular canal and otolith function. Eur. J. Neurol..

[64] Panyakaew P., Anan C., Bhidayasiri R. (2015). Visual deprivation elicits subclinical postural inflexibilities in early Parkinson’s disease. J. Neurol. Sci..

[65] Tramontano M., Bonnì S., Martino Cinnera A., Marchetti F., Caltagirone C., Koch G., Peppe A. (2016). Blindfolded Balance Training in patients with Parkinson’s Disease: a Sensory-Motor Strategy to Improve the Gait. Parkinson’s Disease.

[66] Krauzlis R.J. (2004). Recasting the Smooth Pursuit Eye Movement System. J. Neurophysiol..

[67] R.J. Leigh D.S. Zee The Neurology of Eye Movements (5 Aufl.). 2015 Oxford University Press 10.1093/med/9780199969289.001.0001.

[68] Morimoto H., Asai Y., Johnson E.G., Lohman E.B., Khoo K., Mizutani Y., Mizutani T. (2011). Effect of oculo-motor and gaze stability exercises on postural stability and dynamic visual acuity in healthy young adults. Gait Posture.

[69] Schubert M.C., Migliaccio A.A. (2019). New advances regarding adaptation of the vestibulo-ocular reflex. J. Neurophysiol..

[70] Srulijes K., Mack D.J., Klenk J., Schwickert L., Ihlen E.A.F., Schwenk M., Lindemann U., Meyer M., K.C., S., Hobert, M. A., Brockmann, K., Wurster, I., Pomper, J. K., Synofzik, M., Schneider, E., Ilg, U., Berg, D., Maetzler, W., & Becker, C. (2015). Association between vestibulo-ocular reflex suppression, balance, gait, and fall risk in ageing and neurodegenerative disease: Protocol of a one-year prospective follow-up study. BMC Neurol..

[71] Majcen Rosker Z., Kristjansson E., Vodicar M., Rosker J. (2021). Postural balance and oculomotor control are influenced by neck kinaesthetic functions in elite ice hockey players. Gait Posture.

[72] Cugusi L., Manca A., Dragone D., Deriu F., Solla P., Secci C., Monticone M., Mercuro G. (2017). Nordic walking for the Management of People with Parkinson Disease: a Systematic Review. PM&R.

[73] Bombieri F., Schena F., Pellegrini B., Barone P., Tinazzi M., Erro R. (2017). Walking on four limbs: a systematic review of Nordic walking in Parkinson disease. Parkinsonism Relat. Disord..

[74] Monteiro E.P., Franzoni L.T., Cubillos D.M., De Oliveira Fagundes A., Carvalho A.R., Oliveira H.B., Pantoja P.D., Schuch F.B., Rieder C.R., Martinez F.G., Peyré-Tartaruga L.A. (2017). Effects of Nordic walking training on functional parameters in Parkinson’s disease: a randomized controlled clinical trial. Scand. J. Med. Sci. Sports.

[75] Tschentscher M., Niederseer D., Niebauer J. (2013). Health Benefits of Nordic walking. Am. J. Prev. Med..

[76] Correia A., Pimenta C., Alves M., Virella D. (2021). Better balance: a randomised controlled trial of oculomotor and gaze stability exercises to reduce risk of falling after stroke. Clin. Rehabil..

[77] Hadian M., Raji P., Abasi A., Hoseinabadi R., Baghestani A. (2018). Evaluation of the effect of Vestibular Exercises on Dizziness and Postural Control in Parkinson patients. Journal of Modern Rehabilitation.

[78] Janmohammadi S., Haghgoo H.A., Farahbod M., Overton P.G., Pishyareh E. (2020). Effect of a visual tracking intervention on attention and behavior of children with attention Deficit Hyperactivity Disorder. J. Eye Mov. Res..

[79] Hamacher D., Herold F., Wiegel P., Hamacher D., Schega L. (2015). Brain activity during walking: a systematic review. Neurosci. Biobehav. Rev..

[80] Passos-Monteiro E., Schuch B., F., T. Franzoni, L., R. Carvalho, A., A. Gomeñuka, N., Becker, M., Rieder, C. R. M., Andrade, A., G. Martinez, F., S. Pagnussat, A., & A. Peyré-Tartaruga, L. (2020). Nordic walking and Free walking Improve the Quality of Life, Cognitive Function, and Depressive Symptoms in individuals with Parkinson’s Disease: a Randomized Clinical Trial. Journal of Functional Morphology and Kinesiology.

[81] Peterson D.S., Horak F.B. (2016). Neural Control of walking in people with Parkinsonism. Physiology.

[82] Schröder R., Kasparbauer A.-M., Meyhöfer I., Steffens M., Trautner P., Ettinger U. (2020). Functional connectivity during smooth pursuit eye movements. J. Neurophysiol..

[83] Marx E., Deutschländer A., Stephan T., Dieterich M., Wiesmann M., Brandt T. (2004). Eyes open and eyes closed as rest conditions: Impact on brain activation patterns. Neuroimage.

[84] Ricciardi L., Ferrazzano G., Demartini B., Morgante F., Erro R., Ganos C., Bhatia K.P., Berardelli A., Edwards M. (2016). Know thyself: Exploring interoceptive sensitivity in Parkinson’s disease. J. Neurol. Sci..

[85] Binda P., Kurzawski J.W., Lunghi C., Biagi L., Tosetti M., Morrone M.C. (2018). Response to Short-Term Deprivation of the Human Adult Visual Cortex Measured with 7T BOLD..

[86] Gorges M., Müller H.-P., Lulé D., Pinkhardt E.H., Ludolph A.C., Kassubek J. (2016). The association between alterations of eye movement control and cerebral intrinsic functional connectivity in Parkinson’s disease. Brain Imaging Behav..

[87] Luna B., Velanova K., Geier C.F. (2008). Development of eye-movement control. Brain Cogn..

[88] Cheng F.-Y., Yang Y.-R., Wu Y.-R., Cheng S.-J., Wang R.-Y. (2017). Effects of curved-walking training on curved-walking performance and freezing of gait in individuals with Parkinson’s disease: a randomized controlled trial. Parkinsonism Relat. Disord..

[89] Edwards R.B., Tofari P.J., Cormack S.J., Whyte D.G. (2017). Non-motorized Treadmill running is Associated with Higher Cardiometabolic Demands Compared with Overground and Motorized Treadmill running. Front. Physiol..

[90] Cohen, J. (1988). *Statistical Power Analysis for the Behavioral Sciences* (2. Aufl.). Routledge. https://doi.org/10.4324/9780203771587.

[91] Hopkins W.G., Marshall S.W., Batterham A.M., Hanin J. (2009). Progressive Statistics for Studies in Sports Medicine and Exercise Science. Med. Sci. Sports Exerc..

[92] Salse-Batán J., Sanchez-Lastra M.A., Suarez-Iglesias D., Varela S., Ayán C. (2022). Effects of Nordic walking in people with Parkinson’s disease: a systematic review and meta-analysis. Health Soc. Care Community.

[93] Rizzo J.-R., Hosseini M., Wong E.A., Mackey W.E., Fung J.K., Ahdoot E., Rucker J.C., Raghavan P., Landy M.S., Hudson T.E. (2017). The Intersection between Ocular and Manual Motor Control: Eye–Hand Coordination in acquired Brain Injury. Front. Neurol..

[94] Chao Y.-P., Wu C.W., Lin L.-J., Lai C.-H., Wu H.-Y., Hsu A.-L., Chen C.-N. (2020). Cognitive load of Exercise Influences Cognition and Neuroplasticity of healthy elderly: an Exploratory Investigation. Journal of Medical and Biological Engineering.

[95] Bargary G., Bosten J.M., Goodbourn P.T., Lawrance-Owen A.J., Hogg R.E., Mollon J.D. (2017). Individual differences in human eye movements: an oculomotor signature?. Vision Res..

[96] Lemos J., Pereira D., Almendra L., Rebelo D., Patrício M., Castelhano J., Cunha G., Januário C., Cunha L., Freire A., Castelo-Branco M. (2016). Distinct functional properties of the vertical and horizontal saccadic network in Health and Parkinson’s disease: an eye-tracking and fMRI study. Brain Res..

[97] Ouchi Y., Okada H., Yoshikawa E., Nobezawa S., Futatsubashi M. (1999). Brain activation during maintenance of standing postures in humans. Brain.

[98] Woollacott M., Shumway-Cook A. (2002). Attention and the control of posture and gait: a review of an emerging area of research. Gait Posture.

[99] Huxhold O., Li S.-C., Schmiedek F., Lindenberger U. (2006). Dual-tasking postural control: Aging and the effects of cognitive demand in conjunction with focus of attention. Brain Res. Bull..

[100] Baroncelli L., Lunghi C. (2021). Neuroplasticity of the visual cortex: In sickness and in health. Exp. Neurol..

[101] Gronek P., Haas A.N., Czarny W., Podstawski R., Delabary M.D.S., Clark C.C., Boraczyński M., Tarnas M., Wycichowska P., Pawlaczyk M., Gronek J. (2021). The Mechanism of Physical Activity-induced Amelioration of Parkinson’s Disease: a Narrative Review. Aging Dis..

[102] Reuter I., Mehnert S., Leone P., Kaps M., Oechsner M., Engelhardt M. (2011). Effects of a Flexibility and Relaxation Programme, walking, and Nordic walking on Parkinson’s Disease. Journal of Aging Research.

[103] Dicke U. (2020). Die Funktionelle Neuroanatomie Des Limbischen Systems.

[104] Peters U. (2022). What is the Function of Confirmation Bias?. Erkenntnis.

[105] Mills E.J., Chan A.-W., Wu P., Vail A., Guyatt G.H., Altman D.G. (2009). Design, analysis, and presentation of crossover trials. Trials.

[106] Mak M.K., Wong-Yu I.S., Shen X., Chung C.L. (2017). Long-term effects of exercise and physical therapy in people with Parkinson disease. Nat. Rev. Neurol..

[107] Van Eijkeren F.J.M., Reijmers R.S.J., Kleinveld M.J., Minten A., Bruggen J.P.T., Bloem B.R. (2008). Nordic walking improves mobility in Parkinson’s disease. Mov. Disord..

[108] Pollatsek A., Well A.D. (1995). On the use of counterbalanced designs in cognitive research: a suggestion for a better and more powerful analysis. J. Exp. Psychol. Learn. Mem. Cogn..

[109] Lu C., Louie K.H., Twedell E.L., Vitek J.L., MacKinnon C.D., Cooper S.E. (2022). Overground versus treadmill walking in Parkinson’s disease: Relationship between speed and spatiotemporal gait metrics. Gait Posture.

[110] Michaud L., Richer N., Lajoie Y. (2021). Number of Trials Needed to Assess Postural Control of Young adults in Single and Dual-Task. J. Mot. Behav..

