# Supplementary material for: Effects of a treadmill and oculomotor dual-task intervention vs. -nordic walking on balance in Parkinson’s disease patients − a pilot study
Source: Clin Park Relat Disord. 2025 Sep 8;13:100392. doi: 10.1016/j.prdoa.2025.100392 (PMC12765109; doi:10.1016/j.prdoa.2025.100392)
Supplement: Supplementary Data 1 [file mmc1.docx]

**Table S1.** Motor-cognitive load conditions of static steady-state balance performance

| Nr. | Test conditions | Nr. | Test conditions |
| --- | --- | --- | --- |
| 1. | Standing balance | 2. | Standing balance with eyes closed |
|  |  |  |  |
| 3. | Horizontal smooth pursuit eye movements | 4. | Vertical smooth pursuit eye movements |
|  |  |  |  |
| 5. | Horizontal saccades | 6. | Vertical saccades |
|  |  |  |  |
| 7. | Horizontal rapid eye-head gaze shifts | 8. | Vertical rapid eye-head gaze shifts |
|  |  |  |  |
| 9. | Horizontal gaze stabilization | 10. | Vertical gaze stabilization |
