# Supplementary material for: Effects of a treadmill and oculomotor dual-task intervention vs. -nordic walking on balance in Parkinson’s disease patients − a pilot study
Source: Clin Park Relat Disord. 2025 Sep 8;13:100392. doi: 10.1016/j.prdoa.2025.100392 (PMC12765109; doi:10.1016/j.prdoa.2025.100392)
Supplement: Supplementary Data 2 [file mmc2.docx]

**Table S2.** Oculomotor dual-tasks of the ALT intervention.

| Nr. | Oculomotor  dual-tasks | Involved brain areas and oculomotor function (Heermann, 2017; Leigh & Zee, 2015; Munoz et al., 2022; Nemanich & Earhart, 2016; Trepel, 2022) |
| --- | --- | --- |
| 1. | Horizontal smooth pursuit eye movements | Pons  Superior colliculi  Frontal eye field  Vestibulocerebellum  Lateral + medial rectus muscles |
|  |  |  |
| 2. | Vertical smooth pursuit eye movements | Mesencephalon  Superior colliculi  Vestibulocerebellum  Superior and inferior rectus muscles + superior and inferior oblique muscles |
|  |  |  |
| 3. | Horizontal saccades | Paramedian pontine reticular formation  Thalamus  Frontal eye field  Superior colliculi  Lateral + medial rectus muscles |
|  |  |  |
| 4. | Vertical saccades | Rostral interstitial nucleus of the medial longitudinal fasciculus  Thalamus  Frontal eye field  Superior colliculi  Superior and inferior rectus muscles + superior and inferior oblique muscles |
|  |  |  |
| 5. | Horizontal rapid eye-head gaze shifts | Vestibulocerebellum  Frontal eye field  Mesencephalon  Mm. rectus lateralis + medialis |
|  |  |  |
| 6. | Vertical rapid eye-head gaze shifts | Vestibulocerebellum  Mesencephalon  Superior and inferior rectus muscles + superior and inferior oblique muscles |
|  |  |  |
| 7. | Horizontal gaze stabilization | Nucleus prepositus perihypoglossalis  Superior colliculi  Frontal eye field  Pons  Vestibulocerebellum  Lateral + medial rectus muscles |
|  |  |  |
| 8. | Vertical gaze stabilization | Nucleus prepositus perihypoglossalis  Superior colliculi  Mesencephalon  Vestibulocerebellum  Superior and inferior rectus muscles + superior and inferior oblique muscles |
|  |  |  |
| 9. | Closed eyes | Cerebellum  Somatosensory cortex  Thalamus  Prefrontal cortex  Orbicularis oculi muscle |
